# Advances in the postoperative care of the liver transplant recipient

**DOI:** 10.1097/MCC.0000000000001370

**Published:** 2026-02-09

**Authors:** Neil Campbell, Craig Beattie, Michael A. Gillies

**Affiliations:** a Department of Critical Care; b Scottish Liver Transplant Unit, Royal Infirmary of Edinburgh, Edinburgh, UK

**Keywords:** enhanced recovery, liver transplant, outcomes, personalised medicine

## Abstract

**Purpose of review:**

Survival rates following liver transplantation now exceed 90% at one year. However, the patient group undergoing liver transplantation is increasingly complex, requiring continued focus on improving perioperative care to sustain these survival outcomes. This review highlights recent advances in the postoperative care of the liver transplantation patient.

**Recent findings:**

Modern care integrates Enhanced Recovery After Surgery (ERAS) principles, which emphasise early mobilisation and device minimisation. Risk stratification has become increasingly sophisticated, with frailty and cardiopulmonary exercise testing providing powerful prognostic information; emerging machine learning approaches may further refine personalised risk prediction.

Goal-directed haemodynamic management is advocated, with restrictive fluid strategies and viscoelastic haemostatic assays to minimise transfusion. Advances in graft optimisation have expanded the donor pool: normothermic regional perfusion reduces ischaemic cholangiopathy in donation after cardiac death grafts, while machine perfusion systems show promise in improving early graft function.

Advanced organ support (extracorporeal membrane oxygenation) requires careful graft-conscious management. Infection prevention strategies include tailored prophylaxis approaches. Nutrition and structured prehabilitation/rehabilitation programmes support recovery, reduce complications and address persistent functional deficits.

**Summary:**

Collectively, these developments reflect a shift toward personalised, multidisciplinary postoperative care, aimed at improving both survival and quality of life for liver transplantation recipients.

## INTRODUCTION

Liver transplantation remains the definitive treatment for acute and chronic liver failure and an expanding range of hepatobiliary conditions. Advances in surgical technique, anaesthesia, donor optimisation, and immunosuppression have driven progressive improvements in short-term and long-term survival, with UK 1-year posttransplant survival now exceeding 90% [1]. At the same time, recipients are increasingly older, more comorbid, and transplanted for more complex indications, including acute-on-chronic liver failure and selected oncological diagnoses. Liver transplantation therefore remains a high-risk intervention, requiring coordinated multidisciplinary care and meticulous postoperative management to minimise complications and support recovery. 

**Box 1:**
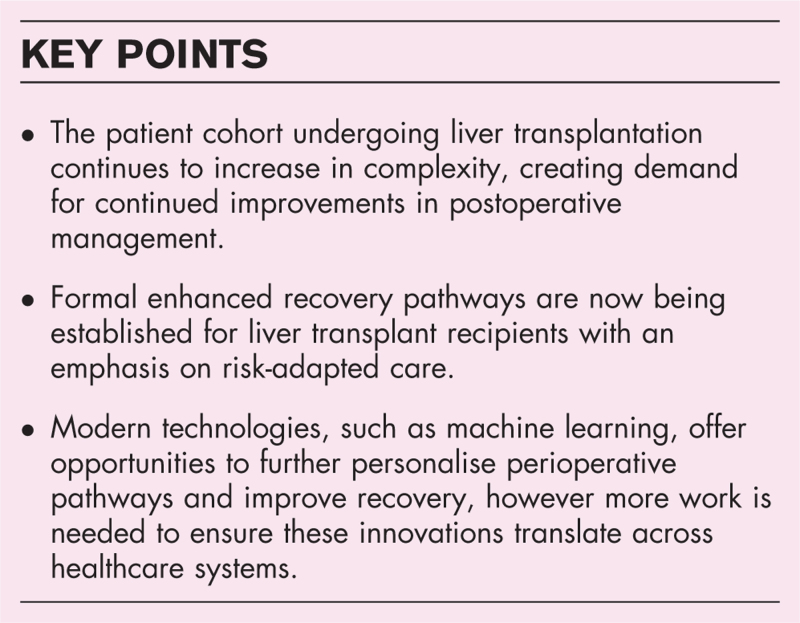
no caption available

## ENHANCED RECOVERY AFTER SURGERY IN LIVER TRANSPLANT

Enhanced recovery after surgery (ERAS) encompasses evidence-based interventions in the pre, intraoperative and postoperative phases of surgery, designed to optimise outcomes and improve patient experience. ERAS protocols have been long established in other surgical specialties; however, their application to liver transplantation has been limited by concerns over patient fragility and procedural complexity.

In 2022, the ERAS Society published the first consensus guidelines on ERAS in liver transplantation. The guidelines outlined 22 perioperative elements across all phases of care [2]. While robust evidence supports some measures, others remain based on limited data, highlighting the need for ongoing prospective validation.

In 2023, the ERAS4OLT.org collaborative, in partnership with the International Liver Transplantation Society (ILTS), expanded this framework through systematic literature review, producing 80 recommendations spanning patient selection, anaesthesia, intensive care and discharge readiness [3^▪▪^].

Central themes across these guidelines include device minimisation, multimodal analgesia, glycaemic control, early physiotherapy and continuous audit to ensure protocol adherence.

A selection of the strongest recommendations from these guidelines is provided in Table 1.

**Table 1:** Postoperative recommendations on enhanced recovery for liver transplant recipients

	Recommendation	Quality of evidence	Grade of recommendation
1.	Early extubation after liver transplantation is associated with improved short-term outcomes and should be performed in most patients.	Moderate	Strong
2.	The use of aspirin is recommended for thromboprophylaxis to prevent hepatic artery thrombosis.	Moderate	Strong
3.	Prophylactic doses of unfractionated heparin or low-molecular-weight heparin should be judiciously considered for recipients to prevent deep vein thrombosis or pulmonary embolism early after liver transplantation.	Moderate	Strong
4.	Both universal prophylaxis and preemptive therapy are recommended for cytomegalovirus prevention in recipients following liver transplantation. The choice of strategy will depend on individual programme resources and experiences, and the serostatus of the donor and recipient.	Low	Strong
5.	Antifungal prophylaxis is recommended for liver-transplant recipients at high risk of developing invasive fungal infections. The choice of drug is dependent on institutional practice.	High	Strong
6.	We propose that abdominal drains, if placed during liver transplantation, should be removed by postoperative day 5, on the basis of quantity and fluid characteristics.	Low	Strong
7.	We recommend that nasogastric tubes can be removed on postoperative day 1 if patients are not ventilated, central lines can be removed once patients are off cardiovascular support, and urinary catheters can be removed once patients are able to mobilise.	Very low	Strong
8.	Tacrolimus, as the standard immunosuppression after liver transplantation, is recommended and can be used in combination with other drugs such as corticosteroids and mycophenolate mofetil, and in association with anti-IL-2 receptor antibody (anti-IL2Ra) induction.	Low	Strong
9.	A low dose or delayed introduction of tacrolimus, in association with corticosteroids, mycophenolate mofetil, and anti-IL2Ra induction, is recommended to reduce acute kidney injury.	Low	Strong
10.	Monitoring of lactate concentrations is recommended given that it represents an early indicator to predict early allograft dysfunction after liver transplantation.	Moderate	Strong

Adapted from [3^▪▪^].

## RISK STRATIFICATION AND PATIENT SELECTION

Identification of patients at greatest risk of complications following liver transplantation allows targeting of ERAS interventions and improved postoperative care. Functional and physiological assessment, cardiopulmonary exercise testing (CPEX) and CT scanning to assess muscle mass are recommended to identify those at risk of increased postoperative complications [4].

Frailty and sarcopenia have emerged as a major determinant of posttransplant trajectory [5]. The Liver Frailty Index (LFI) provides a reproducible, objective measure of functional reserve [6], and comparative studies show that LFI performs reliably in both inpatient and outpatient settings [7]. The EASL Clinical Practice Guidelines on Liver Transplantation (2024) recommend integrating frailty and sarcopenia assessment using muscle indices derived from cross sectional imaging [8]. Standard measurements of frailty are often self-reported or subjective, therefore markers of cellular senescence have been proposed as a biological marker of frailty. Whilst currently only available in a research setting, exploratory studies have linked cellular senescence biomarkers (specifically expression of p16^INK4A^ and p21^CIP1^ mRNA in T cells) with frailty scores and post-liver transplantation length of stay, suggesting potential for enhanced risk stratification [9^▪^].

**FIGURE 1:**
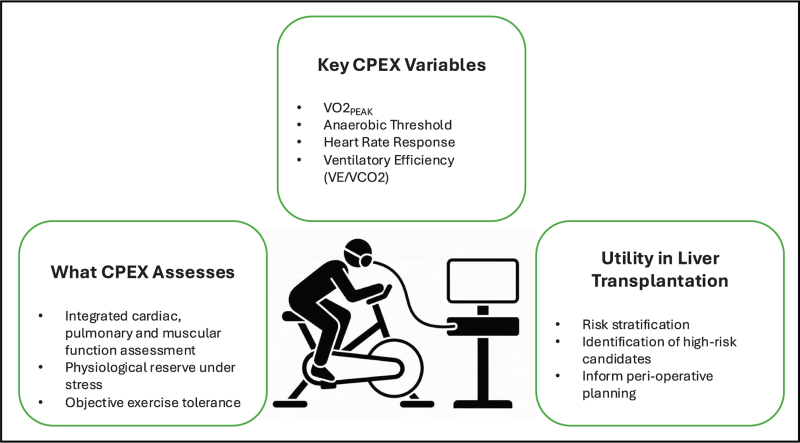
Cardiopulmonary exercise testing in liver transplantation. CPEX is a noninvasive assessment of integrated cardiovascular, respiratory and muscular function performed during incremental exercise. In liver transplant candidates, CPEX provides an objective measure of physiological reserve and supports perioperative risk stratification, guiding the intensity of enhanced recovery and postoperative care pathways.

CPEX remains an established measure of physiological reserve in liver transplantation candidates [10], although its value in high-risk abdominal surgery in general remains uncertain [11]. No universally accepted CPEX thresholds exist specific to liver transplantation, rather its use helps to identify potential high-risk candidates and plan their perioperative care appropriately. The 6-min walk test (6MWT) is an increasingly used, simple test of cardiorespiratory fitness and a recent study in 352 patients awaiting liver transplantation suggested it correlated well with risk of death and duration of hospitalisation. A recent two-centre study of 54 patients undergoing liver transplantation suggested that CPEX combined with 6MWT outperformed dobutamine stress echocardiography for cardiovascular assessment [12]. A summary of the key points of CPEX testing in liver transplantation can be seen in Fig. 1.

There is growing interest in applying machine learning to improve outcomes in liver transplantation. One study compared traditional static risk scores such as Model for End Stage Liver Disease (MELD) with models that incorporate temporal laboratory trends to predict posttransplant mortality [13^▪^]. By modelling laboratory values over time rather than relying on single preoperative measurements, machine learning (ML) approaches demonstrated superior predictive performance. The authors suggest that such continuously updating risk models could enable dynamic, risk-adapted clinical pathways, supporting earlier detection of extubation readiness, proactive prevention of acute kidney injury (AKI), and tailored readmission. However, a multinational analysis using national registries showed that ML or artificial intelligence (AI) models often do not transport across healthcare systems, underscoring the importance of external validation and governance before adopting for local clinical use [14].

## INTRAOPERATIVE AND EARLY POSTOPERATIVE PHASE: ANAESTHETIC AND SURGICAL STRATEGIES

### Haemodynamic monitoring and optimisation

Haemodynamic monitoring and goal-directed therapy (GDT) have long been advocated in high-risk surgery. Pulmonary artery catheters (PACs) and transoesophageal echo are commonly used in liver transplantation [15] alongside other less invasive cardiac output monitors. Use of transpulmonary thermodilution or to deliver GDT has been recommended. Caution around the use of these in the liver transplantation setting is advisable; a recent prospective study found poor correlation between transpulmonary thermodilation using the PiCCO system when compared with PAC [16].

GDT algorithms typically involve the use of cardiac output monitoring to guide fluid therapy, vasoactive drugs and other measures to optimise oxygen delivery to the tissues. The recently published OPTIMISE II trial did not show this approach to be effective at reducing complications in major elective gastrointestinal surgery [17]. EASL 2024 CPGs and a 2024 ERAS-in-LT review still advise GDT approaches to fluid management. This involves using dynamic indices (e.g. stroke volume variation) to guide fluid administration. Excessive fluids are discouraged in liver transplantation to prevent graft congestion, pulmonary complications, and delayed recovery. A systematic review published in 2020 and based largely on observational data suggested that a restrictive approach to fluid management does not reduce the incidence of postoperative AKI, mortality, or other complications [18].

Perioperative haemodynamic instability secondary to cardiac dysfunction, arrythmias, or vasoplegia is common during liver transplantation and may be difficult to manage. Although a mean arterial pressure (MAP) of 65 mmHg is recommended in the intraoperative and postoperative periods, concerns remain that this may be insufficient. The LIVER-PAM study of 174 liver transplantation recipients published in 2025 compared a MAP of 65–70 mmHg to 85–90 on the first postoperative day. No difference in the incidence of AKI was observed and the higher MAP group experienced increased graft rejection [19^▪^]. Current studies are exploring the use Angiotensin II as a second-line vasopressor agent to prevent and treat hypotension in high risk (MELD>25) patients [20].

In summary, a restrictive perioperative fluid strategy, with the avoidance of hypovolaemia and a MAP target of 60–65 mmHg is recommended [3^▪▪^].

### Ventilation and early extubation

Lung-protective ventilation is increasingly protocolised in the immediate postoperative phase. While liver transplantation specific RCTs are lacking, a 2024 meta-analysis of 13 surgical RCTs showed that driving-pressure-guided ventilation reduced postoperative pulmonary complications versus conventional protective ventilation with particularly clear benefit in noncardiothoracic surgery [21]. These findings align with earlier data linking higher driving pressure to increased pulmonary complications, and they are commonly extrapolated to post-liver transplantation ventilation in the absence of trials in this population [21,22].

Early extubation (i.e. within 8 h) has been promoted for liver transplantation recipients for at least a decade. Although prospective trial evidence is not available, observational studies suggest it is feasible and well tolerated in most recipients, including selected high-risk subgroups.

A 2022 systematic review and meta-analysis concluded that early extubation after liver transplantation is associated with shorter ICU stays and low re-intubation rates. Factors significantly associated with deferred extubation in studies included in this review were recipient age, high BMI, ongoing organ failure (e.g. AKI), intraoperative hypotension, long anhepatic time, high intraoperative transfusion requirements, high lactate at the end of surgery, and early graft dysfunction. The evidence from this analysis remains largely observational and future randomised controlled trials (RCTs) with standardised definitions and criteria for early extubation are needed [23]. A 2024 review further highlighted that having the resources to implement an early extubation strategy (shared criteria, physiotherapy on day 1, and explicit escalation plans) was as important as physiological thresholds in achieving successful extubation [24]. In a recent observational study, clinical factors associated with successful early extubation included short anhepatic time, use of epidural analgesia, and the absence of high levels of vasopressor support [25^▪^].

### Patient blood management and haemostasis

Modern patient blood management (PBM) involves anaemia optimisation, iatrogenic blood-loss minimisation, and tolerance of anaemia within physiological bounds, with viscoelastic testing (VET) used to guide targeted blood product use. A 2023 review identified PBM pillars for liver transplantation and highlighted consistent reductions in component transfusion with VET-guided algorithms versus conventional testing across a heterogeneous single-centre series [26]. A 2024 review similarly supports VET-guided transfusion strategies in liver transplantation, noting decreased platelet and FFP exposure without increased bleeding or thrombotic events [27]. Concern about hypercoagulability after bleeding resolves has led to explicit thrombosis surveillance (e.g. doppler studies of the hepatic artery and portal vein) and early pharmacological VTE prophylaxis integrated with VET-guided factor concentrate use [27].

## ORGAN PERFUSION TECHNIQUES

Expanding the liver donor pool to maximise the number of organs available for transplantation includes use of partial grafts and more marginal grafts that may be supported by organ preservation techniques. This has led to the rise of retrieval techniques to improve the number of organs that can be utilised and includes normothermic regional perfusion (NRP), normothermic machine perfusion (NMP), and hypothermic oxygenated perfusion (HOPE/D, HOPE). A systematic review and meta-analysis of NRP in donation after cardiac death (DCD) donors showed that use of NRP significantly reduced ischaemic cholangiopathy, primary nonfunction, and recipient death [28^▪^]. In a 15-centre RCT, whilst not achieving significance, NMP did show a trend towards reduced early allograft dysfunction, particularly in DCD donor organs [29]. In a multicentre phase-III RCT, HOPE reduced the cumulative number of complications over 12 months versus static cold storage, with similar proportions experiencing at least one serious postoperative complication, suggesting a benefit with targeted, rather than universal, use [30]. These technologies have practical implications for postoperative care: more predictable reperfusion physiology, potentially attenuated vasoplegia, and earlier readiness for fast-track pathways.

The optimal organ perfusion strategy is not clearly established. Different centres use different technologies (perfusate, perfusate temperature, pressure, route) and different timing or combination of intervention (donor hospital, during transport, recipient hospital). Questions also remain on optimal biochemical assessment, including liver enzyme analysis, glucose handing, and lactate clearance as best markers of function or nonfunction during machine perfusion [31].

## CRITICAL CARE MANAGEMENT

### Renal support

Post-liver transplantation AKI is a common and prognostically important complication of liver transplantation. In a recent 400 patient living-donor liver transplantation cohort, AKI within occurred the first postoperative week in 62% of patients, and was independently associated with subsequent chronic kidney disease (CKD) [adjusted hazard ratio 1.55, 95% confidence interval (95% CI) 1.12–2.14] [32]. Importantly, if renal function recovered within 90 days, the incidence of long-term CKD was reduced, emphasising the importance of optimising management when it does occur.

Renal replacement therapy (RRT) is often required in the early postoperative course and the indications are multifactorial. In a 2022 single-centre series of over 500 patients, 14.2% received RRT within the first week, and these patients had more severe preoperative liver disease [33]. This report emphasised the importance of minimising haemodynamic disturbance, regional citrate anticoagulation when feasible, and integration with VET-guided haemostasis. As well as preoperative morbidity, postoperative RRT requirement was also associated with greater intraoperative transfusion and lower survival on unadjusted analyses [33].

As discussed above, the current approach to prevention of AKI is vasopressor-first resuscitation, early de-resuscitation, nephrotoxin avoidance, and exposure-guided tacrolimus dosing.

### Extracorporeal membrane oxygenation

Extracorporeal membrane oxygenation (ECMO) post-liver transplantation is rare and outcomes depend on indication, timing, and centre expertise. A contemporary series from a high-volume live-donor liver transplantation programme reported that 0.6% required ECMO perioperatively and survival to discharge was 36.4% overall, consistent with broader perioperative ECMO literature [34]. Di Nardo *et al.* [35] analysed 27 children who required ECMO after liver transplantation using data from the ELSO Registry. Respiratory failure was the most common indication, and overall mortality was high at 63%, with 100% mortality in those placed on ECMO during cardiac arrest. Outcomes were strongly influenced by ECMO-related complications, highlighting that while ECMO can provide rescue support post-liver transplantation, survival remains limited and highly context-dependent [35]. A 2024 narrative review catalogued adult indications and suggested outcomes are better in isolated respiratory failure than multiorgan failure and septic shock, however absolute numbers were small making conclusions difficult [36].

A recent article on the practicalities of ECMO following liver transplantation emphasise graft-conscious cannulation (i.e. cannula placement which avoids portal inflow compromise), meticulous anticoagulation, and close RRT integration [37]. Collectively, the literature supports a cautious, case-by-case approach: ECMO is used rarely, and those requiring it have higher rates of mortality, RRT, and tracheostomy than the broader liver transplantation population [34–37].

## INFECTION PREVENTION

Infection prevention is critical after liver transplantation as the combination of immunosuppression, major surgery, and preexisting organ failure makes recipients highly vulnerable to severe infections. Carugati *et al.* [38] examined 470 adult liver transplant recipients and found that 7.2% developed invasive primary surgical-site infections (IP-SSI) within 90 days. Risk factors included re-transplant, split-liver grafts, Roux-en-Y reconstruction, anastomotic leaks, and need for RRT. Patients with IP-SSI had longer hospital stays and markedly higher mortality at 1 year. IP-SSI are serious postoperative complications driven largely by surgical factors, underscoring the need for targeted prevention and early detection [38].

### Cytomegalovirus

The Fourth International Consensus Guidelines reaffirm D+/R− recipients as the highest risk group and recommend valganciclovir prophylaxis for at least three months (some data suggest six months in very high-risk individuals) or robust PCR-based preemptive strategies. The guidelines also emphasise renal dose adjustment, and caution against nonvalidated low-dose regimens [39].

### *Pneumocystis jirovecii* pneumonia

Co-trimoxazole remains first-line PJP prophylaxis for 6–12 months post-liver transplantation. Where intolerance occurs, a recent multicentre cohort comparing alternative regimens (atovaquone, dapsone) highlighted higher breakthrough rates with these agents and underscored the need to re-challenge co-trimoxazole where feasible [40^▪^].

### Antifungal strategy

A 2024 single-centre cohort study from a high-volume liver transplant programme reported that even with antifungal prophylaxis, invasive Candida surgical-site infections still occurred at appreciable rates. They recommend that centres adopt a multifaceted approach to prevention including identification of patients at risk, prompt treatment of surgical leaks or contamination and surveillance [41]. Complementary analyses further identified specific predictors of invasive surgical-site infection within 90 days posttransplant, informing the development of targeted prevention bundles for high-risk patients.

## NUTRITION

Nutrition can be an overlooked component of recovery. A review of published studies on dietary intake after liver transplantation and found that recipients typically consume around 2000 kcal/day, with diets high in fat and low in fibre, fruits and vegetables. Energy and protein intake are often insufficient in the first month posttransplant but increase thereafter, which may worsen cardiovascular risk. The authors highlight the need for improved nutritional support and long-term dietary counselling in liver transplant recipients [42].

### Rehabilitation and prehabilitation

Rehabilitation is a central pillar of ERAS pathways in liver transplantation, and both the 2024 EASL and 2023 ERAS4OLT guidelines strongly advocate for structured functional optimisation before and after surgery. Modern prehabilitation integrates exercise training, nutritional optimisation, and psychological support, which have been linked to fewer postoperative complications and shorter hospital stays. The ongoing EXALT phase 2b trial is evaluating a home-based, remotely supervised exercise and motivational programme for liver transplantation candidates and recipients, aiming to improve functional capacity, quality of life, and recovery trajectories [43]. Postoperatively, early mobilisation within 24 h is associated with reduced pulmonary and thromboembolic complications and a shorter length of stay and relies on coordinated delivery of daily physiotherapy, clear activity milestones, and proactive nursing involvement [2,3^▪▪^].

## CONCLUSION

**FIGURE 2:**
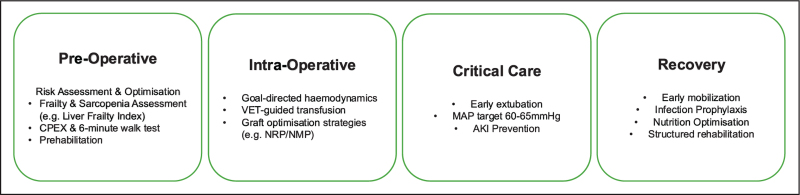
Overview of the modern perioperative care pathway for liver transplantation. Care is increasingly structured around Enhanced Recovery After Surgery (ERAS) principles, incorporating pretransplant risk stratification, graft-conscious intra-operative management, early ICU optimisation, and structured rehabilitation to improve survival and functional outcomes.

Postoperative care following liver transplantation has advanced considerably, driven by developments in ERAS frameworks, refined monitoring strategies, evolving perfusion technologies, and multidisciplinary postoperative pathways. Despite significant improvements in survival, recipients remain vulnerable to complications linked to frailty, graft quality, haemodynamic instability, infection, and renal dysfunction. Evidence consistently supports personalised, risk-adapted care emphasising early mobilisation, physiological optimisation, infection prevention, and sustained rehabilitation. Figure 2 provides an overview of each stage of the perioperative pathway. As ML-driven prediction tools, perfusion technologies, and tailored monitoring strategies mature, they offer the potential for increasingly precise and individualised care pathways. The ongoing challenge is ensuring that these advances translate into reliable, resource-supported, and consistently delivered improvements in postoperative outcomes and quality of life for liver transplant recipients.

## Acknowledgements

*None.*

### Financial support and sponsorship

*None.*

### Conflicts of interest

*There are no conflicts of interest.*

## REFERENCES AND RECOMMENDED READING

Papers of particular interest, published within the annual period of review, have been highlighted as:

▪ of special interest
▪▪ of outstanding interest
